# Could Delayed Primary Epstein–Barr Virus Infection Contribute to the Rising Incidence of Type 1 Diabetes?

**DOI:** 10.1002/rmv.70226

**Published:** 2026-11-10

**Authors:** Cyril Debuysschere, Mahdi Ouafi, Magloire Pandoua Nekoua, Enagnon Kazali Alidjinou, Didier Hober

**Affiliations:** ^1^ Laboratoire de Virologie ULR3610 Univ Lille et CHU de Lille Lille France

**Keywords:** EBV, epidemiology, Epstein–Barr virus, hygiene hypothesis, islet autoimmunity, old friends hypothesis, *orthoherpesviridae*

## Abstract

Type 1 diabetes mellitus (T1DM) incidence has been rising for several decades and varies markedly across countries, a pace and geographical spread that genetic susceptibility alone cannot explain, pointing to a major role for environmental factors. Viruses have long been regarded as triggers of islet autoimmunity, yet recent clinical and experimental observations suggest that Epstein–Barr virus (EBV) may instead exert immunoregulatory effects that could protect against islet autoimmunity. EBV establishes a lifelong infection in approximately 95% of adults worldwide in memory B cells, generally during the first years of life, but the age at primary infection has shifted progressively towards adolescence in high‐income countries, reflecting improved hygiene standards. This review compares the age at primary EBV infection with T1DM incidence across regions and populations that differ in hygiene standards and cultural practices. Populations in which primary infection occurs in early childhood generally show a low incidence of T1DM and a high incidence of nasopharyngeal carcinoma (NPC), an EBV‐induced cancer, whereas the opposite pattern is observed in some populations in which primary infection is delayed; comparable contrasts are found between ethnic groups living within the same country. Delayed exposure to EBV, a virus that has coevolved with the human lineage over millions of years and for which protective effects against islet autoimmunity have been proposed, may therefore contribute, alongside other environmental determinants, to the rising incidence of T1DM, potentially extending the ‘Old friends’ hypothesis to a common virus belonging to the *Orthoherpesviridae* family.

## 1 Viruses and Type 1 Diabetes Mellitus

Type 1 diabetes mellitus (T1DM) is an autoimmune disease characterised by the progressive loss of function of pancreatic islet *β* cells, leading to lifelong dependence on exogenous insulin [1]. This chronic condition mainly affects children and adolescents. Its incidence, which has been rising for several decades, shows marked geographical disparities [2, 3]. The prevalence of T1DM is estimated at approximately 3/1000 in Europe and 4.4/1000 in North America, compared with only 0.6/1000 in Asia and 0.8/1000 in Africa [1].

Although genetic susceptibility is required for disease development, the geographical disparities, the rapid rise in incidence, the seasonality of initial clinical presentations [4], the incomplete concordance among monozygotic twins [1], and observations made in migrant populations [5] all point to a major role for environmental factors in the pathogenesis of T1DM [6]. Although factors such as early exposure to certain foods (eggs, cereals, cow's milk), alterations of the microbiota, and vitamin D deficiency have been suspected [7], viral infections have been the most strongly implicated in the development of islet autoimmunity.

The viral hypothesis posits that exposure to certain viral agents during early childhood or pregnancy may trigger or promote the development of T1DM. It rests on the isolation of viral strains capable of causing pancreatic lesions in murine models, and on the temporal correlation between the clinical onset of T1DM and variations in anti‐coxsackievirus B4 (CVB4) antibody levels observed in affected individuals [8]. Enteroviruses with digestive tropism were the first agents suspected in this context. The role of enteroviruses, including CVB4, was subsequently studied more extensively, and other agents, notably those responsible for respiratory viral infections, have also been implicated [9, 10].

However, the role of viruses in the pathogenesis of T1DM does not appear to be limited to that of triggering or promoting agents. Several observations related to Epstein–Barr virus (EBV) infection in individuals with diabetes suggest that this virus may exert immunoregulatory effects that protect against islet autoimmunity [11]. During primary infection, a transient increase in C‐peptide, increased death of insulin‐specific CD8+ T cells, and an increase in circulating regulatory T cells were reported in a single individual with T1DM [12]. Latent infection with the virus in individuals with T1DM is associated with a more exhausted CD8+ T cell phenotype, a better response to anti‐CD3 therapy, and reduced antigenic response signals compared with uninfected individuals with T1DM [13]. Finally, infection with the virus is also associated with lower production of *β* cell‐specific autoantibodies [14, 15].

EBV is a strictly human virus of the family *Orthoherpesviridae,* subfamily *Gammaherpesvirinae,* which, after primary infection, establishes lifelong persistence as a latent episome in memory B cells. It infects nearly 95% of the world's adult population, generally during the first years of life, although the age at primary EBV infection has become progressively later over recent years [16, 17, 18, 19, 20], particularly in high‐income countries, where infection is now often delayed until adolescence [21]. Asymptomatic infection is the rule when it occurs in early childhood, whereas infection at a later age is associated with a higher risk of infectious mononucleosis, the symptomatic form of primary EBV infection, characterised by the triad of fever, tonsillar pharyngitis, and lymphadenopathy [22].

The increasingly late occurrence of primary EBV infection, attributed to hygiene, the protective effects that have been proposed for this virus in the pathogenesis of T1DM, and the rise in T1DM incidence over several decades raise the question of a relationship between delayed EBV infection and increased T1DM incidence. To address this question, this review compares the age at primary EBV infection with T1DM incidence across regions and populations worldwide that differ in hygiene standards and cultural practices.

## 2 Hygienism, Premastication and Delayed EBV Infection

The insalubrity and recurrent epidemics that accompanied the expansion of large cities during the Industrial Revolution gave rise to hygienism, a movement defined by increased public intervention in matters of sanitation (sewerage, housing, personal hygiene), the benefits of which for public health have been considerable [23]. These principles also extended to childcare practices. Fear of pathogen transmission, together with the industrialisation of infant feeding, altered the practices through which young children are exposed to the saliva of adults, among them premastication (or kiss‐feeding), in which an adult chews food before giving it to a young child. Several anthropological studies attest to the formerly universal character of this practice, which was still common among Inuit at the end of the twentieth century [24] and remains documented in several Indigenous societies of the Pacific [25, 26], as well as in Africa and Southeast Asia [26]. It has not disappeared entirely: its reported prevalence was 69% among caregivers from disadvantaged socioeconomic backgrounds in South Africa in 2011 [27], 26.9% among Chinese children aged 6–36 months in 2018 [28], and 31% among 154 caregivers in US paediatric HIV clinics in 2009–2010 [29].

Although premastication has undergone a major decline, and even disappearance, in some high‐income countries, researchers have questioned the immune benefit of this practice, by analogy with breastfeeding, notably through the transfer of maternal antibodies or of cytokines promoting mucosal immunity or oral tolerance to foods, or through early exposure to the maternal oral microbiota, allowing the development and maturation of the microbiota or of neutralising antibodies [26, 30].

EBV, like the other *Orthoherpesviridae*, is transmitted through saliva. Shedding of the virus in saliva is found in 22%–90% of seropositive individuals [21]. The factors determining the age at primary EBV infection are therefore directly linked to those determining exposure to the saliva of others: young children, lower socioeconomic status, nursery attendance and room sharing, sharing of toys or kitchen utensils, and, of course, premastication. Consequently, the disappearance of these practices, together with improved hygiene conditions in high‐income countries, has substantially altered transmission of the virus to individuals from the earliest age, and is likely to have contributed to the observed shift towards a later age at primary EBV infection [16, 17, 18, 19, 20], the occurrence of infectious mononucleosis cases in these countries [31], and the ethnic differences observed in the prevalence of herpesvirus infections in general [32].

## 3 Regions With Early EBV Infection and Low Reported T1DM Incidence

Several countries in Asia, sub‐Saharan Africa, and South America show a relatively low incidence of T1DM. In these regions, primary EBV infection occurs very early, in a context of conditions of salivary transmission comparable to those that prevailed in high‐income countries before their hygiene transition (Table 1). Some of these countries also show a very high incidence of EBV‐associated cancers, such as nasopharyngeal carcinoma (NPC) [59] or Burkitt lymphoma [60].

**TABLE 1:** EBV seroprevalence and T1DM incidence worldwide.

Country or territory	EBV seroprevalence				T1DM incidence				Interval between sources (y)
	Studied period or year of publication	Age (y)	Seroprevalence (%)	Grade	Studied period year of publication	Age (y)	Incidence (/100,000)	Grade	
East Africa: Kenya Uganda Tanzania	2012 [33] 1992–2008 [34]	0–2 > 1	100 93	Early Early	2010–2016 [35]	< 15	1.8–1.9	Low	1–13
Taiwan	2015 [36]	5–7	88.7	Early	2009–2010 [37]	All	3.3	Low	6
China	2013 [38]	9–13	84.2	Early	2017 [39]	All	3.6	Low	4
	2014 [40]	> 8	90	Early					3
Thailand	2015 [41]	≥ 6	> 95	Early	2015–2020 [42]	0–19	5	Intermediate	3
Iran	2015–2019 [43]	10–14	76.7	Late	2019 [44]	All	11	High	2
Germany	2010–2014 [45]	10–14	∼ 70	Late	2000–2006 [46]	< 15	19.4	High	9
France	2018–2022 [17]	9–14	∼ 60	Late	2015 [47]	0.5–14	19.1	High	5
UK	1994 [48] 2002 [49]	10–14 11–14	50–60 60.4	Late Late	1995–2004 [50]	1–19	21	Very high	3–6
Australia	1975 [51]	11–12	49	Late	2000–2011 [52]	< 14	23.6	Very high	31
Sweden	1967–1997 [53]	9–12	62–64	Late	1983–2007 [54]	0–14	23.6	Very high	13
USA	2003–2010 [55]	9–11	55 (white)	Late	2002–2005 [56]	10–19	26 (white)	Very high	3
			83 (hispanic)	Early			18 (hispanic)	High	
			83 (black)	Early			17 (black)	High	
Saudi Arabia	2018–2023 [57]	14–25	78.2	Late	2017 [58]	All	33.5	Very high	4

*Note:* EBV seroprevalence among the study population: ≥ 80% by approximately 10 years of age, early infection; < 80% by approximately 10 years of age, late infection. T1DM incidence grade (/100,000): < 1, very low; 1–4, low; 5–9.99, intermediate; 10–19.99, high; > 20, very high. Only territories for which both parameters were available are listed. Study periods and age strata differ between sources; values are therefore indicative of overall trends rather than directly comparable. Interval: difference between the midpoints of the two study periods, rounded to the nearest year; a range is given where several serological sources are listed.

In China, T1DM incidence in 2017 was estimated at 3.6 cases per 100,000 inhabitants in the general population [39]. A study published in 2014 showed that EBV seroprevalence reached 90% after 8 years of age [40]. Another study, published in 2013, reported an EBV seroprevalence of 84.2% between 9 and 13 years [38]. NPC incidence in China in 2018 was estimated at 3 per 100,000 inhabitants, approximately seven times higher than in populations of European ancestry [61]. In Taiwan, T1DM incidence around 2010 was estimated at 3.3 per 100,000 inhabitants, and a 2015 study reported an EBV seroprevalence of 88.7% between 5 and 7 years [36]. In Thailand, T1DM incidence between 2015 and 2020 was estimated at 5 cases per 100,000 inhabitants among children and adolescents aged 0–19 years [42]. A 2015 study showed that EBV seroprevalence among Thai individuals exceeded 95% in children older than 6 years [41]. Japan illustrates the recent effect of rising hygiene standards on the age at primary EBV infection. Seroprevalence among children aged 5–7 years fell from more than 80% between 1975 and 1990 to 59% between 1997 and 1999, the authors estimating that it had dropped below 50% by 2006 [20]. In parallel, childhood T1DM incidence rose by 5.3% per year in Oita Prefecture between 1999 and 2021 [62], and by 5.35% per year among children aged 5–9 years in Yamanashi Prefecture between 1986 and 2018, although Japanese incidence currently remains among the lowest in the world [63].

In sub‐Saharan Africa, T1DM incidence is low, even negligible, compared with that of some other countries [64], although it appears to have been increasing in recent years [65]. Several studies have assessed EBV prevalence in the population. A study conducted in Kenya in 2012 in 116 children showed that they generally became infected before the first 15 months of life (on average between 7.28 and 8.39 months) [33]. A study conducted in Ghana in 1978 in 27 children showed that 21 of the 27 children included had been infected before 2 years of age [66]. Finally, a study conducted in Uganda in 2009 showed that among 32 children, approximately 80% were infected by 15 months of age [67]. That study also showed that 84% of the mothers surveyed reported saliva‐sharing behaviours such as premastication [67].

In Argentina, T1DM incidence in 2021 was below 10 per 100,000 inhabitants among children under 15 years of age [64], whereas a seroepidemiological study conducted in 2013 showed that most children are infected with EBV before 3 years of age [68].

## 4 Regions With Delayed EBV Infection and High Reported T1DM Incidence

Some high‐income countries (Europe, North America, Australia) show a very high incidence of T1DM, particularly the Nordic countries. In these countries, where the hygiene transition is long‐standing, primary EBV infection occurs at a later age (Table 1); this shift took place over the twentieth century, in parallel with the rise in T1DM incidence. It is accompanied by a high incidence of infectious mononucleosis, estimated at approximately 60 cases per 100,000 person‐years [69]. A considerably lower incidence of EBV‐associated cancers is also observed in these countries than in Asian countries: NPC incidence is estimated at 0.7 per 100,000 inhabitants in Southern Europe and 0.2 per 100,000 inhabitants in the Nordic countries [70].

In Sweden, T1DM incidence is very high and rose considerably between 2000 and 2010, with more than 40 cases per 100,000 inhabitants among children aged 10–14 years, although this increase appears to have since levelled off [71, 72, 73]. EBV seroprevalence in Sweden has been historically low for many years, at 62%–64% between 1967 and 1997 among children aged 9–12 years [53], and a 2003 study showed that at 4 years of age only 52% of children carried the virus [74]. In France, T1DM incidence in 2015 was estimated at 19.1 cases per 100,000 inhabitants among children under 14 years of age, with an annual increase of approximately 4% [47]. In France, EBV seroprevalence is estimated at 60% among children aged 9–14 years [17], and the age at primary infection rose between 2001 and 2015 [18]. In Germany, T1DM incidence is also high and rising, from 17.1 per 100,000 inhabitants in 1999 to 24.7 per 100,000 inhabitants in 2019 [75]. EBV seroprevalence in the general population appears to reach 90% only at around 20–24 years of age and is estimated to be between 60% and 70% among children aged 5–9 years [45]. In the United Kingdom, T1DM incidence in 2021 was estimated at 28.1 per 100,000 inhabitants among children under 14 years of age [64], whereas EBV seroprevalence reaches 90% between 15 and 19.9 years [76]. In Australia, T1DM incidence estimated between 2002 and 2017 among individuals of European ancestry was 25 per 100,000 inhabitants among children under 15 years of age [77]. A study conducted in 1975 showed a low EBV seroprevalence among children of European ancestry aged 11–12 years in Australia, estimated at 49% [51]. Other countries, such as the United States, New Zealand, and Denmark, also show a high incidence of T1DM, although marked disparities related to the ethnic background of the populations studied are observed.

## 5 Ethnic Disparities

In the United States, a study showed, between 2002 and 2005, a marked disparity in T1DM incidence among children aged 10–19 years, with incidences of 26, 18, and 17 cases per 100,000 inhabitants for White Americans, Hispanic Americans, and Black Americans, respectively [56]. In parallel, a study conducted between 2003 and 2010 showed a much earlier age at primary EBV infection among Black and Hispanic children (89% and 87% of children aged 12–14 years) than among White children (58% of children aged 12–14 years) [55]. These disparities have also been observed in other studies [78, 79, 80]. Alaska Native peoples and people of American Indian descent show a very low prevalence of T1DM compared with the rest of the population, reflected in a very low prevalence of autoantibodies classically associated with T1DM, such as anti‐glutamic acid decarboxylase 65 (GAD65) and anti‐islet antigen 2 (IA2) [81, 82]. Although no robust data on EBV seroprevalence are available in these populations, unpublished data from 1980 suggest that Alaska Native individuals are all seropositive before 4 years of age [83]. Moreover, these populations show a 17.3‐fold higher incidence of NPC than Americans of European descent [84].

T1DM is extremely rare among Greenlandic Inuit populations. A study including 1498 patients with diabetes showed that only 53 had T1DM, corresponding to an estimated prevalence of 0.09% in the general population, compared with 2.59% for type 2 diabetes [85]. In a study of 484 Greenlandic Inuit, the prevalence of anti‐GAD65 autoantibodies was 4.3% among individuals with diabetes and less than 1% among those without diabetes, suggesting that autoimmune diabetes is uncommon in these populations [86]. A study published in 1984 showed that 88% of Inuit children were infected with EBV by the end of their first year of life, and 100% by 3 years of age, compared with 55% and 60%, respectively, among Danish children. Inuit individuals also had higher EBV‐specific antibody titres [87]. The authors further noted that Danes living in Greenland have a higher EBV seroprevalence than Danes living in Denmark [87]. Another study, published in 1985, likewise documented early infection in Inuit children, with EBV seroprevalence estimated at 22% at 6 months, 79% at 2 years, and nearly 100% at 4 years [24]. Finally, Greenlandic Inuit, like Alaska Native peoples, are among the populations with the highest NPC incidence in the world [88], estimated in 1984 at between 8.3 and 11.6 per 100,000 inhabitants, with numerous familial clusters, some of which practiced premastication [89]. This incidence is 20–25 times higher than in Denmark over the same period [87]. Inhabitants of the Arctic Circle therefore appear to depart from the latitude gradient observed for T1DM, which is characterised by increasing incidence with distance from the equator [90].

Similar disparities are observed in the Pacific Islands. In New Zealand, T1DM affects people of European ancestry more than Māori, Asian, or other Pacific peoples: in 2021, children of European origin aged 0–14 years had a 2.6‐fold higher risk of T1DM than Māori children [91]. In parallel, Māori are 4 times more likely to develop NPC than people of European ancestry, and Pacific peoples 21 times more likely [92]. T1DM incidence in Fiji between 2001 and 2012 was estimated at 0.93 cases per 100,000 inhabitants across all groups, with a striking difference according to ancestry: 87% of T1DM cases were found among Indo‐Fijians, although this group makes up only 37% of the population [93]. In Papua New Guinea, T1DM incidence in 2001 was 0.08 cases per 100,000 inhabitants, with a prevalence of 0.28 per 100,000 inhabitants among those under 15 years of age (2001) [94]. For comparison, incidence in Australia over the same period was estimated at 21.6 cases per 100,000 inhabitants [95].

A study carried out on samples collected in 1960 examining EBV seroprevalence in Papua New Guinea showed that 90% of children were infected before 4 years of age. The authors attributed this early infection to the multiple caregivers of each child, and also to the practice of premastication [96].

## 6 Limitations of the Comparative Approach

Across the regions and populations presented here, a relationship between a late age at primary EBV infection and T1DM incidence may be suspected, but the comparisons on which it rests are ecological: the two parameters were measured in separate populations, by separate investigators, and juxtaposed at the level of countries or ethnic groups rather than individuals. They are not contemporaneous, age strata differ between sources, seroprevalence was not always assessed in the age range most informative for the timing of primary infection, and all‐age incidence cannot be compared directly with childhood incidence. Serological assays, antigen targets and sampling frames are heterogeneous and may bias seroprevalence estimates in either direction. Case ascertainment is also uneven: where diagnostic capacity, access to care and registry coverage are limited, as in sub‐Saharan Africa, Papua New Guinea and other Pacific territories, recorded T1DM incidence probably underestimates true incidence, so part of the contrast with high‐income countries may reflect detection rather than disease.

Therefore, no inference about individual risk can be drawn from such comparisons, and the association described is compatible with, but does not establish, a relationship between the timing of primary EBV infection and the subsequent development of islet autoimmunity.

## 7 Age of EBV Primary Infection, NPC and T1DM

The epidemiological data presented indicate that, in regions or populations characterised by an early age at EBV infection, T1DM incidence is generally lower, whereas NPC incidence is higher. This is particularly true of populations such as Alaska Native peoples and Greenlandic Inuit [86, 88]. EBV is the etiological agent of nearly all NPC: it is estimated to contribute to 95% of NPC cases in endemic areas [97]. An association between an early age at EBV infection and an increased risk of NPC has already been suggested: in Sweden, markers of early exposure to the virus, such as a large sibship or older siblings, were shown to increase the risk of NPC while reducing the risk of developing infectious mononucleosis (IM), which is characteristic of late infection [98]. Two further studies reported a similar trend without reaching statistical significance [98]. Accordingly, if early EBV infection were to favour the development of NPC while reducing the risk of T1DM, the inverse association observed between the incidences of these two diseases would be expected (Figure 1). Nevertheless, some countries of the Maghreb, for example, appear to show a high incidence of both diseases, a reminder that the pathophysiological processes underlying them are multifactorial (genetic and environmental) and not related solely to the age at EBV infection.

**FIGURE 1:**
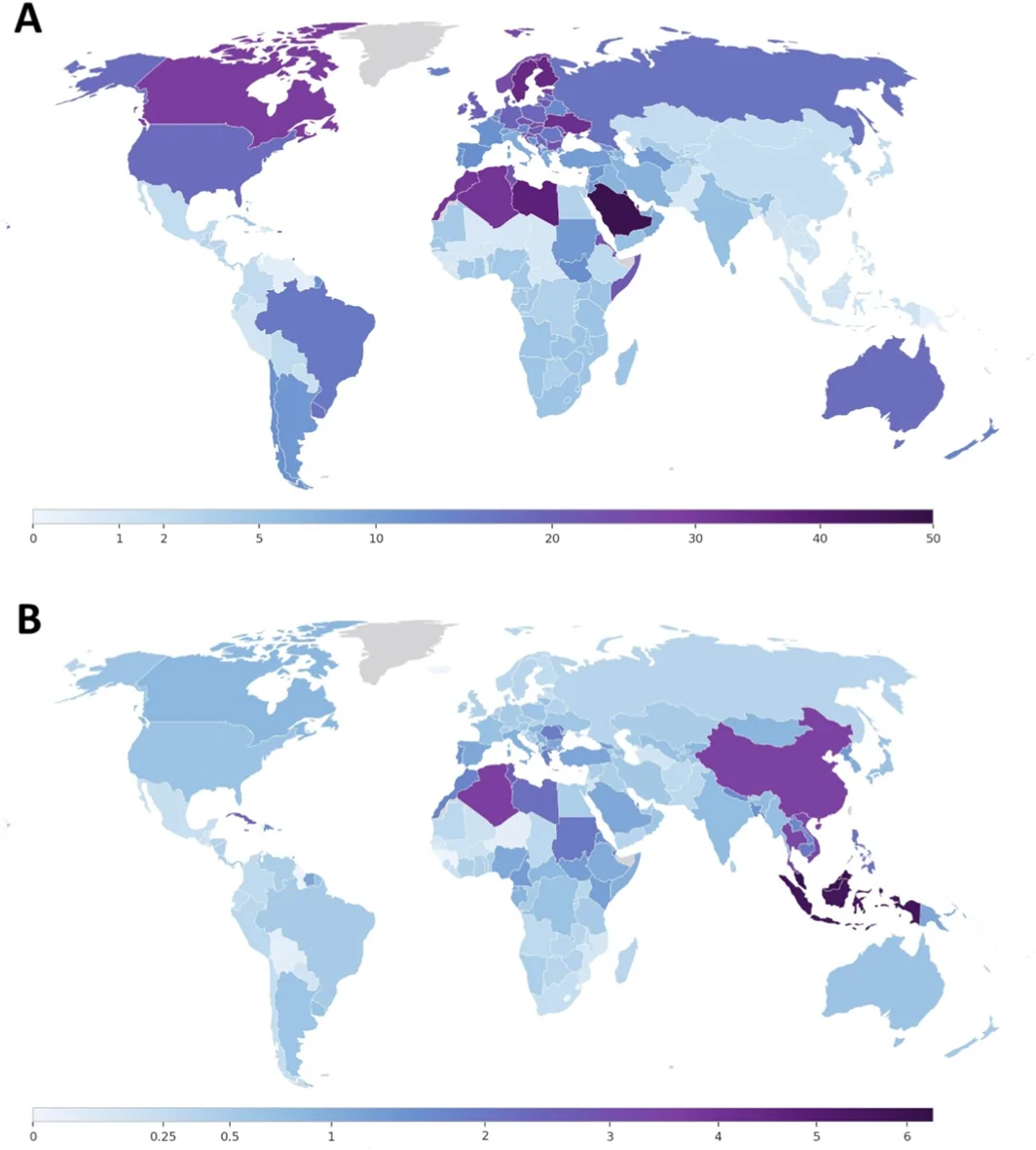
Inverse geographical distribution of type 1 diabetes and nasopharyngeal carcinoma incidence in 2024. (A) T1DM incidence. (B) NPC incidence. For each country, the estimated number of incident cases was divided by the total population for the same year (world bank) and expressed per 100,000 inhabitants (colour scale). Estimated incident cases were obtained from the T1D index (breakthrough T1D, ISPAD, Life for a Child, IDF) for T1DM and from the global cancer observatory (IARC) for NPC. European and North American countries, as well as several Gulf countries, show high T1DM and low NPC incidence, whereas several East and South‐East Asian countries show the opposite pattern. Some North African countries are an exception, with high incidence of both diseases. T1DM, type 1 diabetes mellitus; NPC, nasopharyngeal carcinoma.

## 8 EBV, the Hygiene Hypothesis and the Old Friends Hypothesis

Several non‐exclusive elements may account for the geographical pattern described above, beyond EBV delayed infection. Genetic susceptibility is difficult to invoke on its own, since the haplotypes conferring risk differ between populations [99]. The rise has moreover taken place within a few decades, a time scale incompatible with a change in genetic background: high‐risk HLA genotypes are less frequent in recent cohorts than in older ones [100], and the rise is driven by cases carrying lower‐risk genotypes [101]. Latitude‐based explanations are also incomplete. Ultraviolet B exposure and vitamin D status have been proposed to account for the latitude gradient of T1DM, but Sardinia, at approximately 40°N, reports an incidence (45 per 100,000) close to that of Finland (64.2 per 100,000) at some 62°N [102], while the Arctic populations discussed above combine extreme latitude with a very low prevalence of islet autoimmunity. Finland and the adjacent Russian Karelia, whose inhabitants share a common ancestry, are at the same latitude and have comparable frequencies of HLA risk genotypes, yet differ approximately sixfold in childhood T1DM incidence, a contrast attributed to standard of living and microbial exposure [103], both of which are also determinants of the age at primary EBV infection.

Socioeconomic conditions (standard of living, household composition, infant feeding practices, antibiotic use, and access to care) shape microbial exposure during the first years of life and may contribute to geographical variations in T1DM incidence. Gut dysbiosis has been proposed as a potential contributor to T1DM pathogenesis. For example, *Bifidobacterium longum* subsp. *infantis* is prevalent in infants from low‐ and middle‐income countries but is rarely detected in high‐income countries [104]. In one Singapore birth cohort, only 4.4% of infants carried this subspecies [105]. Other geographical differences have been reported in Northern Europe, with Karelian infants enriched in *Bifidobacterium* and Finnish and Estonian infants in *Bacteroides.* [106] Differences in the immunogenicity of lipopolysaccharide (LPS) from different bacterial species have been proposed as a potential mechanism underlying these observations. However, the role of the gut microbiota in T1DM development remains uncertain. In TEDDY, no microbial taxon was found to predict islet autoimmunity or T1DM [107], and a recent systematic review did not support the loss of *Bifidobacterium* as a key factor in disease development [108]. Helminth infections are also less prevalent in high‐income countries. Although experimental helminth infection can protect against diabetes in non‐obese diabetic (NOD) mice [109], evidence for a protective effect against T1DM in humans remains limited. Similarly, the age at primary infection with EBV and other saliva‐transmitted viruses varies according to socioeconomic conditions and patterns of early‐life social contact.

Several clinical and experimental observations made in individuals with T1DM are compatible with a protective effect of EBV infection in this disease, although they are few in number and largely observational [11]. In a single T1DM individual, EBV primary infection was accompanied by a transient rise in C‐peptide, depletion of insulin‐specific CD8+ T cells and expansion of circulating regulatory T cells [12], while a cohort study showed that latent infection is associated with a regulated/exhausted CD8+ T cell phenotype, dampened antigen receptor signalling and enhanced responsiveness to anti‐CD3 therapy [13], and infection overall (whether primary or latent) is associated with lower production of *β* cell‐specific autoantibodies [14, 15].

A very early exposure to immunoregulatory effects of EBV might reduce the risk of T1DM, while promoting the development of NPC when combined with other genetic and environmental factors (consumption of salted fish, tobacco, and alcohol) (Figure 2A). The data discussed in this review might therefore reflect a direct link between late EBV infection and a higher incidence of T1DM (Figure 2B).

**FIGURE 2:**
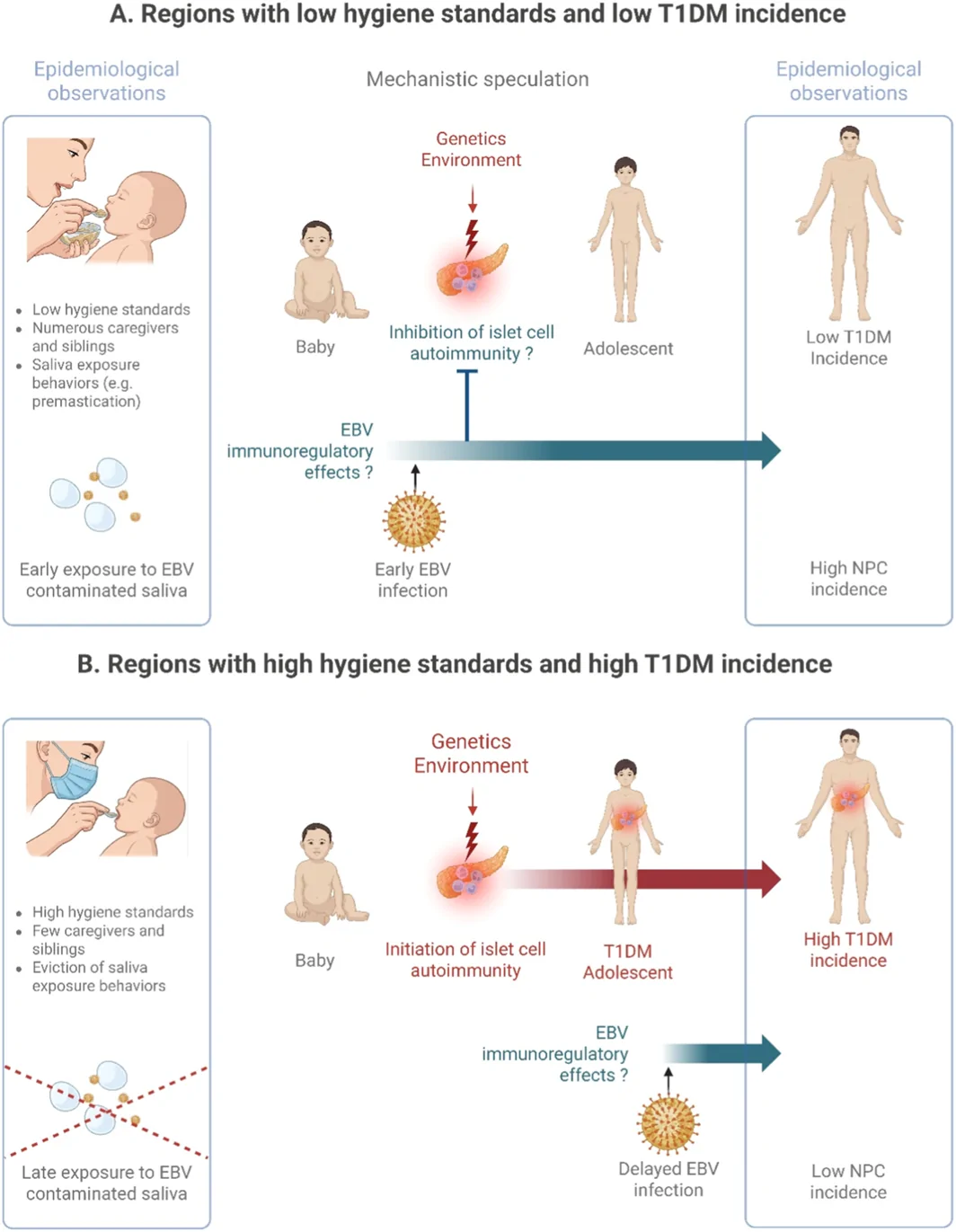
Proposed hypothesis linking the age at primary EBV infection and the incidence of T1DM. (A) In settings with low hygiene standards, where salivary exposure occurs early, primary EBV infection takes place in infancy. The immunoregulatory mechanisms accompanying the establishment of lifelong latency would then be present before the usual window of initiation of islet autoimmunity, and could limit it; the same early infection has been associated with an increased risk of NPC. (B) In settings where early salivary exposure is reduced, primary infection is delayed, and these mechanisms would be established only after the period during which islet autoimmunity is initiated. Genetic susceptibility and environmental factors other than EBV may contribute to both conditions and are not detailed here. EBV, Epstein–Barr virus; NPC, nasopharyngeal carcinoma; T1DM, type 1 diabetes mellitus. Created with BioRender.

These observations can be framed within the hygiene hypothesis, which suggests that the increase in certain allergic and autoimmune diseases (including T1DM) in industrialised countries is related to reduced exposure to infections during childhood. The ‘Old friends’ hypothesis refines it by proposing that what matters above all is reduced exposure to microorganisms that have coevolved with humans over thousands of years and that are able to stimulate the immunoregulatory mechanisms of the human immune system: the induction and maintenance of regulatory T cells and the production of anti‐inflammatory cytokines. Accordingly, current lifestyles in some populations would lead to reduced or delayed exposure to these microorganisms, resulting in impaired maturation of the immune system and altered immune tolerance [110, 111, 112].

EBV belongs to the genus *Lymphocryptovirus*, whose phylogeny broadly tracks that of its primate hosts. EBV clusters in a subclade estimated to have diverged from an Old World monkey lineage some 12 million years ago. The virus has thus coevolved with the human lineage since its origin [113]. Unlike the exposures usually considered under the hygiene hypothesis, this ancient companion has not disappeared: it remains nearly universal and establishes lifelong persistence within memory B cells, central players of the adaptive immune system. What has changed is not exposure to the virus itself, but the timing of primary infection. Given this ancient relationship, the recent shift towards a later age at primary infection with the virus could fall within the framework of the ‘Old friends’ hypothesis and entail significant immunological consequences, among them the rise in T1DM incidence in many countries.

EBV is not the only saliva‐transmitted herpesvirus whose age at primary infection has risen with hygiene standards. Cytomegalovirus (CMV) follows a closely parallel socioeconomic and geographical gradient [114], and a prospective study in children at genetic risk found that early childhood CMV infection, while not affecting the appearance of islet autoantibodies, was associated with slower progression to clinical T1DM [115]. The hypothesis proposed here might therefore not be restricted to EBV, and could extend to other saliva‐transmitted *Orthoherpesviridae*.

To date, no prospective study has directly tested the relationship proposed here for EBV. Such studies will be necessary to push forward this hypothesis and to clarify the potential role of Epstein–Barr virus infection in the incidence of T1DM.

## Author Contributions

**Cyril Debuysschere:** conceptualisation, methodology, investigation, formal analysis, data curation, writing – original draft, writing – review and editing, visualisation. **Mahdi Ouafi:** writing – review and editing. **Magloire Pandoua Nekoua:** writing – review and editing. **Enagnon Kazali Alidjinou:** writing – review and editing. **Didier Hober:** supervision, validation, writing – review and editing, funding acquisition. All authors have read and approved the final version of the manuscript.

## Conflicts of Interest

The authors declare no conflicts of interest.

## Data Availability

Data sharing not applicable to this article as no datasets were generated or analysed during the current study.
